# Role of the ITAM-Bearing Receptors Expressed by Natural Killer Cells in Cancer

**DOI:** 10.3389/fimmu.2022.898745

**Published:** 2022-06-10

**Authors:** Hakim Medjouel Khlifi, Sophie Guia, Eric Vivier, Emilie Narni-Mancinelli

**Affiliations:** ^1^ Aix-Marseille Université, Centre National de la Recherche Scientifique (CNRS), Institut National de la Santé et de la Recherche Médicale (INSERM), Centre d’Immunologie de Marseille-Luminy (CIML), Marseille, France; ^2^ Innate Pharma Research Laboratories, Marseille, France; ^3^ APHM, Hôpital de la Timone, Marseille-Immunopôle, Marseille, France

**Keywords:** NK cells, ITAM, cancer, NCR, activating receptors

## Abstract

Natural Killer (NK) cells are innate lymphoid cells (ILCs) capable of recognizing and directly killing tumor cells. They also secrete cytokines and chemokines, which participate in the shaping of the adaptive response. NK cells identify tumor cells and are activated through a net positive signal from inhibitory and activating receptors. Several activating NK cell receptors are coupled to adaptor molecules containing an immunoreceptor tyrosine-based activation motif (ITAM). These receptors include CD16 and the natural cytotoxic receptors NKp46, NKp44, NKp30 in humans. The powerful antitumor NK cell response triggered by these activating receptors has made them attractive targets for exploitation in immunotherapy. In this review, we will discuss the different activating receptors associated with ITAM-bearing cell surface receptors expressed on NK cells, their modulations in the tumor context and the various therapeutic tools developed to boost NK cell responses in cancer patients.

## NK Cells: An Overview

NK cells belong to the ILC family, which encompasses several other subsets: helper-like ILC (ILC1 to 3) and lymphoid tissue inducer cells (LTi). NK cells are endowed with cytolytic functions, whereas helper-like ILC are characterized mainly by their cytokine secretion profiles, which mirror those of helper CD4^+^ T cells. ILC1 produce IFN-γ type 1 cytokine, ILC2 produce type 2 cytokines, such as IL-5 and IL-13, and ILC3 secrete IL-17 and IL-22 type 3 cytokines (1).

In humans and mice, NK cells circulate in the peripheral blood and infiltrate most organs, including the spleen, liver, lymph nodes and bone marrow. They are also located in the barrier organs of the body, such as the intestines and lungs (2). The NK cell compartment is heterogeneous. Human NK cell subsets are generally defined on the basis of their cell surface expression of CD56 and CD16 (FcγRIIIA) and lack of the T cell receptor complex (CD3/TCR). Human NK cells have been split into two main subsets: CD56^dim^ CD16^+^ NK cells and CD56^bright^ CD16^−^ NK cells, which are found in the bloodstream and most organs (3, 4). According to their transcriptomic profiles, these two subsets are found in the blood and spleen of humans and conserved in mice at homeostasis. They were defined as NK1/CD56^dim^-like and NK2/CD56^bright^-like cells (5).

CD56^dim^ CD16^+^ cells are cytotoxic and express killer-cell immunoglobulin-like receptors (KIRs), whereas CD56^bright^ CD16^−^ NK cells are less mature, produce larger amounts of cytokines and chemokines and are more prone to proliferate under cytokine stimulation (6). Several studies have suggested that CD56^dim^ CD16^+^ cells can differentiate from CD56^bright^ CD16^−^ cells (7, 8). We recently showed that an additional NK0/CD56^bright^-like subset displays specific enrichment in the expression of genes associated with the NK cell precursor (NKP) signature. This tissue-specific subset differs from NK2/CD56^bright^-like cells in its higher levels of expression of CD52, CD127 and a lack of CD160. A bioinformatic algorithm inferring developmental trajectories from scRNAseq data showed that NK0/CD56^bright^ CD160^−^-like cells could differentiate into both NK1/CD56^dim^ and NK2/CD56^bright^ CD160^+^-like cells, but that NK1 and NK2 cells did not appear to be developmentally related under physiological conditions (9).

In addition, some individuals harbor a particular population of NK cells, “adaptive NK”, characterized by the cell surface activating receptor NKG2C and the maturation marker CD57 in humans (10). This subset results from the expansion and persistence after contraction of NK cells following cytomegalovirus (CMV) infection. Adaptive NK cells can be recovered from both humans and mice, and have been detected following the activation of NK cells following hantavirus infection (11), herpes simplex virus 2 infection (12) or influenza vaccination in humans (13).

Another population, cytokine-induced memory-like (CIML) NK cells, has also been described in both humans and mice (14–16). Following prior activation with an IL-12/IL-15/IL-18 cytokine cocktail, the capacity of these cells to produce IFN-γ and granzyme B after restimulation increases.

Thus, several subsets of NK cells have been identified in healthy donors by bulk RNA sequencing (RNAseq), single-cell RNAseq or Cytometry by time of flight (CYTOF). However, it remains to be verified whether all these NK cell populations are indeed present in the majority of individuals. Also, it is of interest to define the heterogeneity of NK cells in several cancer indications.

## Role of NK Cells in Cancer

NK cells are cytolytic and exocytose cytotoxic granules containing both perforin (a membrane-disrupting protein) and granzymes (a family of proteolytic enzymes) at the immunological synapse with target cells, leading to target cell lysis. Activated human NK cells also express death-inducing ligands, such as FAS ligand and TNF-related apoptosis-inducing ligand (TRAIL). In addition to their cytotoxic activities, NK cells can produce cytokines, such as IFN-γ and TNF-α. IFN-γ has direct antitumor properties, but also activates myeloid cells and contributes to the shaping of T-cell responses. NK cells can also secrete growth factors (e.g. granulocyte–macrophage colony-stimulating factor) and chemokines (CCL3, CCL4 and CCL5), which participate in the recruitment of dendritic cells in inflamed tissues (17–19).

Many data from *in vitro* studies have shown that NK cells kill cancer cells of different histological origins. *In vivo*, depletion experiments and the use of mouse models with impaired NK cell activity have demonstrated higher rates of tumor growth and metastasis (20, 21). Unfortunately, the depleting antibodies used in these experiments were not specific for NK cells. The anti-NK1.1 antibody can also target ILC1, natural killer T cells (NKT) and activated T cells, and the anti-asialo GM1 serum can also target NKT, some T-cell populations, γδ T cells, basophils, eosinophils and macrophages (22, 23). NKp46 is mostly restricted to NK cells, but it is also expressed by ILC1, a subset of ILC3 and a minority fraction of T cells. As a result, no NKp46-depleting antibody or mouse models with modified *Ncr1* gene (coding for NKp46) expression can specifically target NK cells. In this context, it is, therefore, important to bear in mind that the results obtained with these tools and attributed to NK cells might actually be due to other cell populations, such as ILC1. Along this line, investigations of role of ILC1 in tumor responses are an emerging field of research, and opposite effects have already been reported in the mouse. On the one hand, ILC1 expansion and cytotoxicity are associated with the control of tumor growth in a mouse mammary tumor model (24) and with the limitation of metastatic seeding in the liver for MC38 colon carcinoma cells and Lewis lung carcinoma cells (LLC) (25). On the other hand, NK cell differentiation towards cells with an ILC1-like gene signature has been shown to be associated with uncontrolled melanoma cell metastasis in the lung (26) and chemically induced fibrosarcoma (27, 28). In this last model, intratumoral ILC1 may limit the NK1.1^+^ cell-dependent antitumoral response, or even foster tumor growth (28). These results should, therefore, prompt evaluation of differential tumor infiltration and the role of ILC1 and NK cells in optimizing the harnessing of immunity in the treatment of cancer patients.

In humans, cancer incidence is higher in patients with NK cell deficiencies characterized by an absence of NK cells or poor effector functions due to mutations of the MCM4 or GATA2 genes (29, 30) (see **Table 1**). However, these data should be interpreted with caution as such mutations also affect immune cells other than NK cells; it is thus not possible to establish a formal link between NK cell activity and cancer control based on these findings.

**Table 1:** Clinical evidence for a role of NK cells in controlling cancer.

Evidence	Cancer type	Prognosis	References
Dysfunction in NK cell cytotoxicity	Multiple cancer types	Higher rate of cancer incidence	(31)
NK cell lymphopenia and GATA2 deficiency	Acute myeloid leukaemia	Higher rate of cancer incidence	(30)
NK cell lymphopenia and MCM4 deficiency	Lymphoma	Higher rate of cancer incidence	(29)
NK cell infiltration	Gastric cancer	Better patient prognosis	(32)
	Hepatocellular carcinoma	Better patient prognosis	(33)
	Squamous cell lung cancer	Better patient prognosis	(34)
	Non-small cell lung cancer	Better patient prognosis	(35)
	Head and neck cancer	Better patient prognosis	(36)
	Melanoma	Better patient prognosis	(37)
NK cell gene signature	Neuroblastoma	Better patient prognosis	(38)
Presence of NKp46 transcripts	Melanoma	Better patient prognosis	(39)

A few studies have monitored the cytotoxic activity of blood NK cells and the risk of developing cancer (40, 41). In an 11-year follow-up survey conducted in the Japanese general population, the group of patients with low levels of NK cell cytotoxic activity had a higher risk of cancer (31). Along the same lines, low NK cell counts, rather than CD4^+^ or CD8^+^ T cells in peripheral blood are associated with lower overall survival in patients with follicular lymphoma (42).

Many studies have also reported defective blood NK cell function in patients with solid tumors. Our analysis of predefined NK and CD8^+^ T-cell signatures in multiple public transcriptome data from the TCGA database, which includes over 10,000 tumor samples corresponding to 33 different cancer types, showed that most tissues infiltrated by NK cells were also infiltrated by T cells. Contrary to the findings for ILC1, the NK signature was strongest in hematopoietic cancers (acute myeloid leukemia (AML) and B-cell lymphoma), followed by solid cancers, including kidney renal clear cell carcinoma, testicular germ cell tumors, mesothelioma (a very rare form of cancer affecting the membranes lining internal organs, such as the pleura, peritoneum and pericardium), thymoma, cervical squamous cell carcinoma, endocervical adenocarcinoma, lung cancer and gastric cancers (esophagus, stomach, pancreas) (43) (see **Table 1**).

Even if there is no formal proof that NK cells are involved in immunosurveillance, NK cells have been shown to have antitumor effects in clinical studies. In several xenograft models in which human cancer cells are implanted in immunodeficient mice, the injection of human NK cells controls tumor elimination (44, 45). Solid evidence for the targeting of human tumors by NK cells in patients has been provided by studies of allogeneic hematopoietic stem cell transplantation (HSCT), in which donor NK cells expressing inhibitory KIRs mismatched with the patient’s cells for MHC class I (MHC-I) molecules because they recognized recipient myeloid leukemia cells as foreign cells (46).

In solid tumors, NK cell infiltration seems to be essential for a robust immune checkpoint blockade (ICB) response (47). Interestingly, transcriptomic analysis of patient samples corresponding to gastric (32), neuroblastoma (38), hepatocellular carcinoma (33), non-small cell lung cancer (NSCLC) (48), head and neck (36) and melanoma (37) tumors have revealed a correlation between NK cell infiltration and better patient outcomes regardless of treatment. Furthermore, NK cell infiltration in solid tumors, as monitored by immunohistochemical analyses of renal cell carcinoma (49), breast cancer (50), adenocarcinoma lung cancer (51), squamous cell lung cancer (34) and NSCLC (35) patients, is associated with better overall survival.

Based on all of this evidence, the targeting of NK cells as been proposed as a means of improving immunotherapies for cancer control, at the forefront of cancer research.

## NK Cell Activation

NK cells gauge their environment by integrating signals delivered through ligands binding to inhibitory and activating germline-encoded receptors. The long intracellular part of inhibitory receptors contains a highly conserved signaling motif, the immunoreceptor tyrosine-based inhibitory motif (ITIM) defined by the consensus sequence S/I/V/LxYxxI/V/L. When this inhibitory receptor is triggered, the ITIM is tyrosine phosphorylated, leading to the recruitment of the SH2 domain (Src Homogy domain 2)-containing protein phosphatases SHP-1/2 or phosphatidylinositol phosphatase SHIP (52). These phosphatases dephosphorylate key signaling molecules involved in the activation pathways, thereby inhibiting cell activation. This inhibitory signal results principally from the interaction of inhibitory receptors, such as KIR in humans and Ly49 in mice, with physiological levels of MHC-I molecules. The CD94/NKG2A heterodimer is another major inhibitory receptor, expressed by around half the NK cells in human and mice; it is capable of recognizing non-classical MHC-I molecules, such as human leukocyte antigen (HLA)-E in humans and Qa-1^b^ in mice (53, 54).

**Figure 1:**
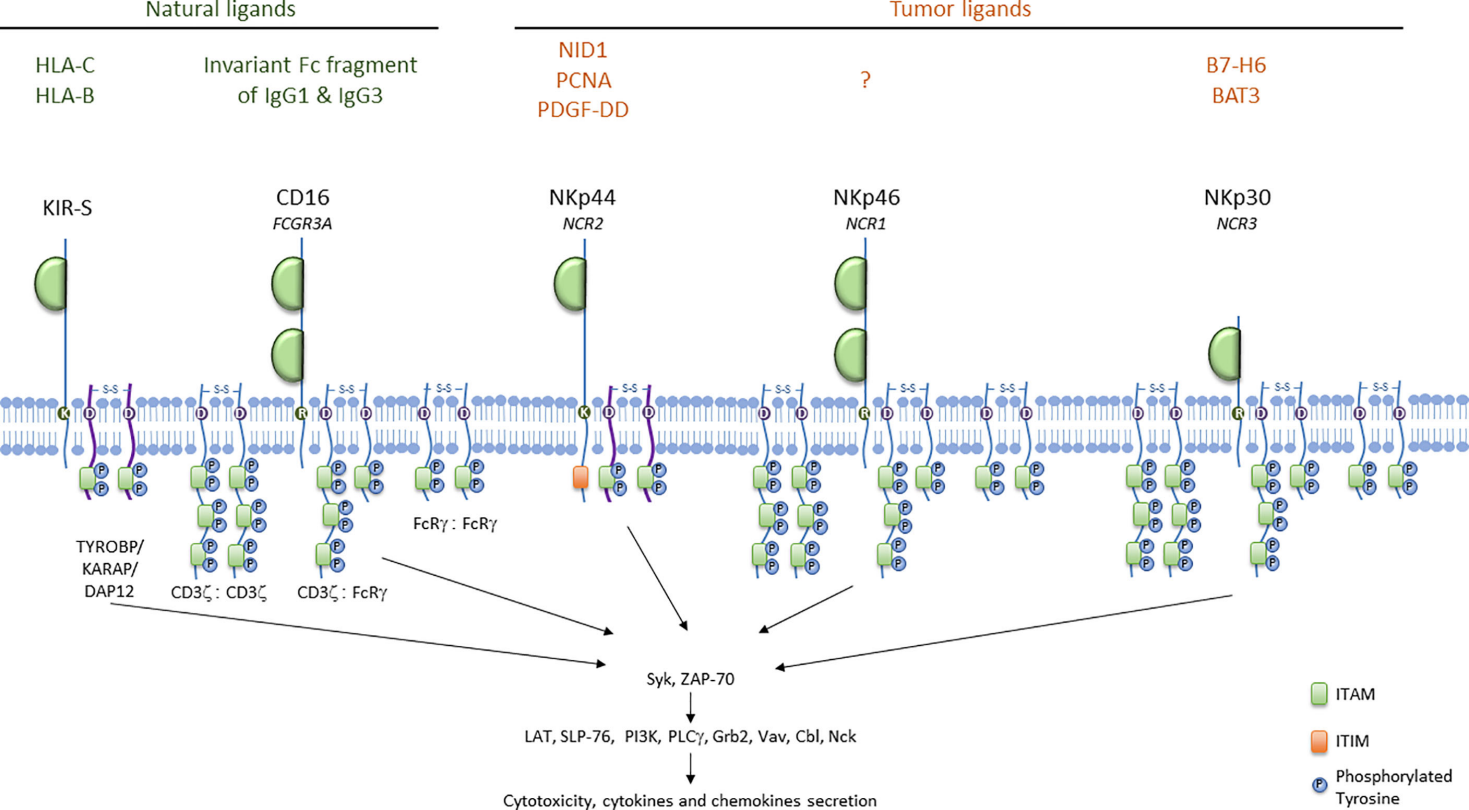
ITAM-bearing receptors in humans. ITAM-bearing receptors in humans are represented at the membrane associated either with CD3ζ or with FcRγ as homo- or heterodimers. ITAM motifs are represented with the proteins involved in the activation signal cascade. Natural ligands for KIR-S and CD16, and tumor ligands for NCRs are indicated.

NK cell activation results from the engagement of activating receptors, such as the activating isoforms of KIRs and Ly49, the natural cytotoxicity receptors (NCRs), the SLAM (signaling lymphocyte activating molecule)-related receptors, NKG2D and CD16, leading to NK cell activation through the initiation of different signaling pathways (55). Activating receptors can be classified into three groups: receptors associating with adaptors bearing the immunoreceptor tyrosine-based activation motif (ITAM) (activating KIRs, CD16 and NCRs), NKG2D and other receptors signaling *via* different pathways (CD2, 2B4, DNAM-1 and NKp80). NKG2D associate with DAP10 in humans but with DAP12 (also known as TYROBP/KARAP) in mice. CD16, NKp46 and NKp30 are coupled to CD3ζ and FcRγ as homo- or heterodimers, whereas NKp44 and KIR-S signal through DAP-12 (**Figure 1**). CD3ζ, FcRγ and DAP12 harbor ITAM motifs defined by the consensus sequence YxxL/Ix_(6-8)_YxxL/I (56, 57). Cell-surface receptor aggregation induces the recruitment of Src protein tyrosine kinases, which phosphorylate the ITAM, leading to the recruitment and activation of the tandem SH2 domain–containing Syk and ZAP-70 tyrosine kinases. This cascade of events leads to the recruitment and phosphorylation of multiple signaling molecules (LAT, SLP-76, PI3K, PLCγ, Grb2, Vav, Cbl, Nck) and the expression of subsequent effector functions (52). Adaptor molecules differ in the number of ITAMs. FcRγ and DAP12 each contain only one ITAM, whereas CD3ζ has three ITAM motifs. The CD16 Fc receptor, NKp46 and NKp30 can, therefore, theoretically signal *via* the phosphorylation of up to six ITAMs, whereas KIR-S and NKp44 can deliver activating signals *via* the phosphorylation of only two ITAMs (55).

## Cell Surface Receptors Associated With ITAM-Bearing Polypeptides in Humans

### Activating KIRs

*KIR* genes are located in the leukocyte receptor complex on chromosome 19. The *KIR* gene family encompasses 15 genes and 2 pseudogenes. The KIR nomenclature indicates the structure and the function of the receptor, and take into account the nucleotide sequence similarity among the different *KIR* family members (17). Thus, the nomenclature indicates the numbers of extracellular domains (2D or 3D), and provides information on the length of the cytoplasmic tail (long L or short S). In general, activating receptors have a short cytoplasmic fragment and possess in their transmembrane region, of a charged lysine residue that allows their association with the DAP12 bearing the ITAM motif. Only the KIR2DL4 receptor can conduct both activating and inhibitory signals. KIR genotypes are divided into A and B haplotypes: haplotype A contains only one activating receptor 2DS4 while haplotype B contain different combinations of the activating genes KIR2DS1, -S2, -S3, -S5 and KIR3DS1. Haplotype B can contains from one to five activating KIR receptors. KIR ligands are HLA class I, HLA-C and HLA-B as indicated in **Figure 1**. Additionally, recent work has shown that KIR2DS2^+^ NK cells can respond to different malignant cell lines *in vitro* through the interaction of KIR2DS2 with a β2-microglobulin-independent ligand (58).

Upon engagement by their ligands, activating KIRs signal *via* DAP12 and promote NK cell functions. Correlation between activating KIR and cancer prevalence is still unclear, but it has been shown that KIR2DS1, 2DS3, 2DS5, 3DS1 are associated with overall survival in gastric cancer (59). However, the presence of KIR2DS2, regardless of the HLA-C genotype, is associated with neuroblastoma (60).

### CD16

The *FCGR3A* gene encodes the CD16 receptor. This receptor consists of two extracellular Ig domains, a short cytoplasmic tail and a transmembrane domain (**Figure 1**). It associates with FcRγ and CD3ζ homodimers or heterodimers in humans and with FcRγ homodimers in mouse NK cells (56, 57). CD16A is a low to intermediate-affinity glycoprotein transmembrane receptor for the Fc domains of IgG1 and IgG3 expressed by NK cells, whereas the isoform CD16B is mainly expressed by neutrophils. CD16 engagement initiates antibody-dependent cell-mediated cytotoxicity (ADCC), and CD16A expression levels are positively correlated with the ADCC potency of NK cells (61, 62). Upon CD16A engagement, tumor cells can be killed by the release of cytotoxic granules. Also, NK cells produce proinflammatory cytokines, such as IFN-γ, and chemokines, which lead to the recruitment and activation of tumor-infiltrating immune cells, contributing to tumor cell killing (63–71). CD16 triggering also affects NK cell survival, as CD16 ligation facilitates proliferation and sustained growth of IL-2-activated human NK cells (72). However, CD16 ligation also promotes NK cell death (73).

Two polymorphic variants of CD16A have been described resulting from a single nucleotide polymorphism (SNP). These allelic variants differ in their affinity for IgG with a KD of 0.75 µM for CD16A-158V and 5 µM for CD16A-158F, and this can affect the efficacy of tumor-targeting therapeutic mAbs (74). Patients homozygous for the higher-affinity CD16A variant with follicular lymphoma (FL) (75), non-Hodgkin lymphoma (76), CRC (77), or breast cancer (78) have a better clinical outcome after treatment with anti-tumor mAbs than patients with the low-affinity CD16 allele (see **Table 2**). This correlation has been observed in many, but not all studies (106–110), suggesting that other parameters may also be involved, such as variability in copy numbers of *FCGR3A* transcripts (61). In addition to recognizing IgG, CD16A has been shown to mediate spontaneous cytotoxicity of melanoma cells (111) by associating with CD2 in *cis* to recognize CD58 as a ligand (112).

**Table 2:** Dysregulation of NK cell surface receptor associated with ITAM-bearing polypeptides in cancer.

Activating Receptor	Analysis	Evidence	Cancer Type	Prognosis	References
CD16	Flow cytometry & Transcriptomic analysis	CD56^dim^CD16^-^ NK cells are the dominant subset in the tumor microenvironment	Melanoma	Better patient outcome	(79)
	Flow cytometry	Low levels of CD16 expression at the cell surface of CD56^+^ NK cells	Breast cancer	?	(78)
			Colorectal cancer	?	(80)
			Ovarian cancer	?	(81)
			Squamous cell carcinoma of the head and neck	Worse patient outcome	(82)
	Sequencing	Patients homozygotes for the CD16A-158V treated with therapeutic mAbs	Follicular lymphoma	Better patient outcome	(75)
			Non-Hodgkin lymphoma	Better patient outcome	(76)
			Colorectal cancer	Better patient outcome	(77)
			Breast cancer	Better patient outcome	(78)
NKp46	Transcriptomic analysis	Low levels of NKp46 transcripts in stage IV patients	Melanoma	Worse patient outcome	(39)
	Flow cytometry	Low levels of NKp46 at the cell surface of NK cells	Acute myeloid leukaemia	Worse patient outcome	(83–85)
			Cervical cancer	Worse patient outcome	(86)
	Flow cytometry	Normal levels of NKp46 at the cell surface of NK cells	Breast cancer	?	(87)
			Liver cancer	?	(87)
			Head and neck cancer	?	(87)
			Metastatic melanoma	?	(87)
			Lung cancer	?	(87)
			Kidney cancer	?	(87)
NKp30	Protein assay, Flow cytometry & Transcriptomic analysis	Soluble B7-H6 serum levels are correlated with NKp30 downregulation	High risk neuroblastoma	Worse patient outcome	(88)
			Pediatric neuroblastoma	Worse patient outcome	(88)
	Flow cytometry	Low levels of NKp30 at the cell surface of NK cells	Acute myeloid leukaemia	Worse patient outcome	(89)
			Breast cancer	Worse patient outcome	(90)
			Chronic lymphocytic leukaemia	Worse patient outcome	(91)
	Transcriptomic analysis	Overall reduction in NKp30 mRNA transcript levels	Melanoma	Higher levels of NKp30 mRNA isoform C transcripts are correlated with worse prognosis	(39)
	Transcriptomic analysis	High levels of NCR3 transcripts	Head and neck cancer	Better patient outcome	(92)
			Lung adenocarcinoma	Better patient outcome	(92)
			Cutaneous melanoma	Better patient outcome	(92)
			Sarcoma	Better patient outcome	(92)
	Protein assay	High levels of BAT3 in the serum	Hodgkin lymphoma	Worse patient outcome	(93)
			Chronic lymphocytic leukaemia	Worse patient outcome	(94)
	Transcriptomic analysis	BAT3 is overexpressed in tumor cells	Hepatocellular carcinoma	Worse patient outcome	(95)
	Transcriptomic analysis & Protein assay	Levels of soluble BAT3 are correlated with downregulation of NKp30 and mRNA transcripts	Gastrointestinal stromal tumor	Worse patient outcome	(96)
	Sequencing	Presence of BAT3 rs3117582 SNP	Non-small cell lung lancer	Association of BAT3 rs3117582 SNP with an increased risk of developing non-small cell lung lancer	(97)
	IHC	High expression of B7-H6 in the tumor	Hepatocellular carcinoma	Worse patient outcome	(98)
			Ovarian cancer	Worse patient outcome	(99)
	Transcriptomic analysis & Protein assay	High levels of soluble B7-H6 in the serum and high expression of B7-H6 in the tumor	Gastrointestinal tumor	Worse patient outcome	(96)
			Neuroblastoma	Worse patient outcome	(100)
NKp44	Transcriptomic analysis & IHC	High expression of NID1 at the tumor cell surface	Glioma	Worse patient outcome	(101)
			Head and neck squamous cell carcinoma	?	(101)
	Protein assay	High levels of NID1 in the serum	Non-small cell lung cancer	?	(102)
			Ovarian cancer	Worse patient outcome	(103)
	Transcriptomic analysis	High expression of PDGF-DD in tumor cells	Low grade glioma	Better prognosis	(104)
			Bladder cancer	Better prognosis	(105)

Unlike other activating receptors expressed by NK cells, CD16A displays a downregulation of its surface expression after triggering, by cleavage and shedding induced by the ADAM17 metalloprotease (113–117). However, ADAM17 deficiency in humans does not affect NK cell effector activities (118). ADAM-17 is overexpressed in several human cancers, such as non-small cell lung cancer (119) and gastric carcinoma (120, 121), and this overexpression is associated with poor patient survival. CD16A levels on the surface of NK cells are downregulated in the tumor microenvironment of colorectal carcinoma (80), ovarian carcinoma (81), head and neck cancers (82), breast cancer (122) and melanoma (79), contributing to NK cell dysfunction.

### NKp46

The NCR family has three members: NKp46, NKp44 and NKp30. NKp46 (or CD335, NCR1), is the only NCR conserved across mammalian species; it is expressed by all mature NK cells, ILC1 (123), a subset of ILC3, and Tγδ cells (124). NKp46, encoded by the *NCR1* gene, is a 46 kDa type 1 transmembrane protein from the immunoglobulin (Ig) superfamily characterized by two immunologublin-C2-like extracellular domains connected by a stalk domain to a transmembrane domain and a short cytoplasmic tail. NKp46 is associated with CD3ζ and/or FcRγ at the cell membrane (125) (**Figure 1**).

NKp46 recognizes several microbial ligands, a surface protein on healthy pancreatic β cells and the soluble complement factor P (124). *In vitro*, NK cells can kill different types of tumor cells *via* NKp46, but the identity of the ligand involved remains unknown. *In vivo*, genetic deficiencies of NKp46 in mice impair the clearance of subcutaneous PD1.6 T lymphoma (126), RET melanoma tumors (127) and B16 melanoma metastases in the lung (127–129). Moreover, the overexpression of an NKp46 transgene has been shown to enhance the clearance of melanoma metastases in the lung (130). Enhanced NKp46 signaling has been shown to elicit IFN-γ secretion and to increase fibronectin deposition in the tumor, altering the architecture of the solid tumor and decreasing the formation of melanoma metastases (39).

NKp46 expression at the cell surface is downregulated on NK cells/ILC1 from patients with acute myeloid leukemia, this phenotype being reversible upon complete remission (83–85) (see **Table 2**). Patients with cervical cancer also present a decrease in NKp46 expression on the surface of NK cells/ILC1 (86). Still, the level of surface expression of NKp46 in breast, liver, head and neck, metastatic melanoma, lung and kidney cancer patients remains quite similar to the one of healthy donors and was not correlated with NK cell dysfunction (87).

### NKp30

NKp30 (or CD337, NCR3) is a 30 kDa protein expressed by all mature human NK cells (131), ILC2, and tonsil-derived ILC3. This surface receptor consists of an extracellular IgV domain and a hydrophobic transmembrane domain with a charged arginine residue capable of associating with the adaptor proteins CD3ζ and/or FcRγ (132) (**Figure 1**).

NKp30 has six variants (NKp30a, NKp30b, NKp30c, NKp30d, NKp30e and NKp30f), due to alternative splicing. NKp30a, NKp30b and NKp30c have a V-type Ig-like extracellular domain, whereas NKp30d, NKp30e and NKp30f have a different C-type Ig extracellular domain. It has been reported that the triggering of the NKp30a and NKp30b variants promotes the production of IFN-γ, whereas cells transfected with the NKp30c variant produce high levels of immunosuppressive IL-10 (133). This difference may reflect the strong association of the NKp30a and NKp30b variants with CD3ζ and FcRγ, whereas NKp30c associates very little with these adaptors (133, 134), but the relevance of these findings remains to be understood.

NKp30 recognizes microbial and tumor-derived ligands. The tumor ligands it recognizes include nuclear HLA-B-associated transcript 3 (BAT3), a protein chaperone released from tumors (135) and reported to trigger NK cell activation. Conversely, soluble BAT3 has been detected in the serum of patients with Hodgkin lymphoma and chronic lymphocyte leukemia (93, 94), and has been shown to suppress NK cell activation. BAT3 also promotes melanoma metastasis (136, 137) and has been identified as a biomarker for poorer patient survival in hepatocellular carcinoma (95), gastrointestinal stromal tumor (96) non-small cell lung cancer (97) and lung adenocarcinoma (138) patients (see **Table 2**).

NKp30 binds to B7-H6, a B7 family member. Membrane expression of B7-H6 renders cancer cells sensitive to NK cell-mediated cytolysis (139). Conversely, NK cells chronically stimulated with soluble forms of B7-H6 resulting from metalloprotease-mediated shedding may display a downregulation of NKp30 expression contributing to tumor immune escape (140). Soluble B7-H6 has been detected in the serum of patients with hepatocellular carcinoma (98) or gastrointestinal tumors (96) and in peritoneal fluid from patients with ovarian cancer (99). High levels of soluble B7-H6 are also associated with bone marrow metastasis and the chemoresistance of neuroblastoma cells (88, 100).

Overall, increases in the level of NCR3 transcripts have been reported to be associated with better patient survival in the context of head and neck cancer, lung adenocarcinoma, cutaneous melanoma and sarcoma (92). NKp30a and NKp30b are associated with better survival and prognosis for gastrointestinal stromal tumors (133, 141), hepatocellular carcinoma (142) and pediatric neuroblastoma (88), whereas morbidity is higher in patients expressing predominantly the NKp30c isoform. NKp30 protein levels at the surface of NK cells are lower in patients with breast cancer (90) and chronic lymphocytic leukemia (91). The monitoring of NKp30 expression could thus be of interest to predict NK cell dysfunction. As such, NKp30 is downregulated in acute myeloid leukemia and NKp30 status has been proposed as an early prognostic biomarker identifying patients at intermediate risk with a poor prognosis (89).

### NKp44

NKp44 is an activating receptor expressed by activated human NK cells, tonsil NCR^+^ ILC3s and plasmacytoid dendritic cells in humans, but not in mice. NKp44 is a 44 kDa protein consisting of an extracellular immunoglobulin V-like domain linked by a stalk domain to a transmembrane domain connected to an intracellular moiety (132) (**Figure 1**). At the membrane, NKp44 associates with the adaptor protein DAP12.Three NKp44 mRNA splice variants (NKp44-1,−2,−3) have been reported, each endowed with different signaling capabilities based on the presence (NKp44-1) or absence (NKp44-2 and−3) of an ITIM in their cytoplasmic tail. Initially described as non-functional (143), this ITIM may, in some cases, transduce an inhibitory signal (144). NKp44-1 expression has been associated with poor survival in AML patients (145).

NKp44 recognizes various tumor ligands, including the ‘proliferating cell nuclear antigen’ (PCNA) (146), platelet-derived growth factor D (PDGF-DD) (147), nidogen 1 (148).

PCNA is a nuclear protein involved in regulating DNA replication, DNA repair, and cell cycle progression; it may be overexpressed in cancer cells, promoting tumor survival and malignancy (149). The NKp44/PCNA axis inhibits NK cell-mediated cytotoxicity and IFN-γ secretion *via* the ITIM motif of the NKp44-1 variant (145).

NKp44 also recognizes the soluble PDGF-DD, a member of the PDGF family corresponding to the active processed form of PDGF-D (150, 151). Remarkably, NKp44/PDGF-DD interaction promotes the secretion of IFN-γ and TNF-α by IL-2-activated NK cells, but not cytotoxicity, limiting tumor cell growth *in vitro* and tumor spread in a transgenic NCR2 mouse model (147). PDGF-DD is secreted by various tumors and promotes cell proliferation, stromal cell recruitment, angiogenesis, epithelial-mesenchymal transition and metastasis (152, 153) (see **Table 2**).

Barrow and coworkers recently identified transcriptional signatures unique to PDGF-DD-activated NK cells and established the abundance of this signature in The Cancer Genome Atlas (TCGA) low-grade glioma (LGG) and bladder cancer (BLCA) datasets with CIBERSORT. They found that tumors of patients enriched in PDGF-DD-activated NK cell or memory CD8^+^ T-cell phenotypes were associated with a more favorable prognosis in LGG, but with a poor prognosis in BLCA (104, 105).

Nidogen 1 (NID1) is an extracellular matrix protein (148) that promotes epithelial-mesenchymal transition (EMT) and metastasis in ovarian cancer. NID1 increases the adhesion of ETV5-overexpressing endometrial cancer cells to the extracellular matrix (ECM) and promotes their proliferation and migration. The NKp44/NID1 axis does not induce classical NK cell activation, but it does inhibit the IFN-γ production induced by PDGF-DD following NKp44 engagement. Soluble NID1 levels are high in non-small cell lung cancer (102), ovarian (103), head and neck squamous cell carcinoma and glioma (101) (see **Table 2**).

Finally, NKp44 can also recognize a subset of HLA-DP molecules, depending on HLA-DP allotype (HLA-DP401), this interaction being further modulated by the peptide presented by HLA-DP molecules (154). The NKp44/HLA-DP401 axis induces the production of IFN-γ by NK cells. Tumor cells exposed to IFN-γ express HLA-II (155). The NKp44/HLA-DP401 axis may, therefore, contribute to the favorable prognosis of certain tumors with high levels of HLA-II expression (156).

## Harnessing NK Cell Activating Receptors

The mechanisms underlying the therapeutic effect of antibodies used in clinical practice depend on the cell types infiltrating the tumor and the type of FcγR they express. Unlike myeloid cells and B cells, which also display inhibitory FcγR isoforms, NK cells express only the CD16A activating receptor for IgG, and are therefore particularly responsive to therapeutic mAb-dependent activation. Several studies have reported that increases in the numbers of intratumoral and circulating NK cells are associated with favorable outcomes of treatment with anti-HER2-mAbs, anti-EGFR-mAb (cetuximab) and anti-CD20-mAb (obinutuzumab and rituximab) for breast cancer, head and neck squamous cell carcinoma, follicular lymphoma and diffuse large B-cell lymphoma (157–162). Furthermore, the Treg-mediated suppression of ADCC is correlated with lower clinical efficacy in cetuximab-treated HNSCC patients (163).

The first clinical studies of bispecific NK cell engagers (BiKEs) date back to the 1990s. A F(ab’)2 format molecule was generated to target CD16 and NK cells and CD30 as the tumor antigen (Tag), to treat end-stage Hodgkin’s disease (164–166). Unfortunately, this treatment was highly toxic. Novel BiKEs have been generated in recent years, to link scFvs against an NK-cell receptor (CD16, NKp46, NKp30 or NKG2D) and a Tag (BCMA, CD19, CD20, CD30, CD33, CD38, CD133, EPCAM and GPC3) (166–173). The engineering of the Fc portion of tumor-targeting mAbs makes specific targeting of the FcRγ subtype possible. Specific genetic mutations and glycoengineering can be used to increase the affinity of the IgG for CD16. The anti-CD20 mAb obinutuzumab differs from rituximab in having a defucosylated Fc region, increasing CD16 binding affinity (174–177). In combination with chemotherapy, the antitumor efficacy of obinutuzumab was superior to that of rituximab, with the elicitation of stronger ADCC due to its binding to both CD16A-158V and 158F, indifferently, in patients suffering from chronic lymphocytic leukemia (CLL) or follicular lymphoma (178–181)

Another way to improve the responsiveness of NK cells is to stimulate them with cytokines. The IL-12/IL-15/IL-18 inflammatory cytokine cocktail can confer memory-like features on murine and human NK cells in the absence of an antigen (14, 16, 182). Heteromeric IL-12/IL-15/IL-18/CD16scFv fusion proteins were found not to increase NK cell reactivity over that achieved with IL-12/IL-15/IL-18, and were, therefore, not developed any further. Furthermore, the reactivity of CIML NK cells against HuT-78 lymphoblasts in the presence of AFM13 (CD16/CD33) is moderately higher than that of freshly isolated NK cells (183). However, TriKEs composed of one scFv against CD16, another against CD33 and a human IL-15 crosslinker promote NK cell effector functions, and also increase NK cell expansion and persistence *in vivo* in mouse preclinical models of AML and ovarian cancer (184, 185). Thus, co-engagement of the ITAM and JAK/STAT pathways renders the NK cell response more potent. The humanized TriKE CD16/IL-15/CD33 is currently under evaluation in a phase I/II trial for the treatment of high-risk myelodysplastic syndromes, refractory/relapsed AML and advanced systemic mastocytosis (NCT03214666).

NKp46 appears to be a particularly interesting potential target, as it is persistently expressed on tumor-infiltrating NK cells, whereas NKp30, CD16 and NKG2D are frequently downregulated. NKp46 is also more NK-cell specific, as NKG2D is widely expressed in T cells and can cause severe T cell-associated toxicities (70). Bispecific NK cell engagers against NKp46 and tumor antigen GPC3 are being developed (186). Trispecific NK cell engagers targeting both NKp46 and CD16 on NK cells together with a Tag, named ANKET (Antibody-based NK cell engager therapeutics), have been generated. Bispecific NKp46-Tag activate NK cells, but trispecific ANKET have greater potency for the activation of NK cells *in vitro* and for increasing the number of NK cells at the tumor site, thereby providing a higher degree of tumor growth control in solid and invasive mouse tumor models. The co-engagement of a larger number of NK cell activating receptors therefore increases efficacy. Trispecific ANKET can control tumor development in preclinical models of B cell lymphoma (87). In pediatric B-cell acute lymphoblastic leukemia, the trispecific ANKET targeting NKp46, CD16 and CD19 has been shown to have greater cytotoxicity against BCP-ALL cell lines and to lead to an increase in the percentage of IFN-γ^+^ NK cells (187). Finally, tetraspecific ANKET combining NKp46/CD16/CD20 and IL-2 are now being generated and are displaying strong preclinical antitumor efficacy against CD20^+^ tumor cells and an ability to increase NK cell proliferation (Demaria et al., unpublished data).

## Concluding Remarks

High levels of NK cell infiltration have been associated with a better prognosis, at least for some tumors. However, the tumor microenvironment may affect NK cell invasion of the tumor mass and may also have suppressive activities against NK cells. It is thus of interest to favor NK cell infiltration of tumors and to boost NK cell functions in the tumor bed. The antitumor response of NK cells is effectively stimulated by tumor-targeting mAbs, through NCRs and CD16 triggering. Current research is aiming to promote NK cell survival/proliferation, recruitment, and persistence at tumor sites. Future studies will aim to potentiate these immunotherapies by combining them with immune checkpoint inhibitors, chemotherapy or drugs targeting the immunosuppressive tumor microenvironment.

## Author Contributions

All authors contributed to gathering of data, writing, editing, and revising of the manuscript.

## Funding

The EV laboratory at CIML and Assistance-Publique des Hopitaux de Marseille is supported by funding from the European Research Council (ERC) under the European Union’s Horizon 2020 research and innovation program (TILC, grant agreement No. 694502 and MInfla-TILC, grant agreement No. 875102 - MInfla-Tilc), the Agence Nationale de la Recherche including the PIONEER Project (ANR-17-RHUS0007), MSDAvenir, Innate Pharma and institutional grants to the CIML (INSERM, CNRS, and Aix-Marseille University) and to Marseille Immunopole. The funder was not involved in the study design, collection, analysis, interpretation of data, the writing of this article or the decision to submit it for publication. HMK is recipient of a PhD grant from ‘Institut Cancer Immunologie’.

## Conflict of Interest

EV is an employee of Innate Pharma.

The remaining authors declare that the research was conducted in the absence of any commercial or financial relationships that could be construed as a potential conflict of interest.

## Publisher’s Note

All claims expressed in this article are solely those of the authors and do not necessarily represent those of their affiliated organizations, or those of the publisher, the editors and the reviewers. Any product that may be evaluated in this article, or claim that may be made by its manufacturer, is not guaranteed or endorsed by the publisher.
